# Adsorption of Toluene and Paraxylene from Aqueous Solution Using Pure and Iron Oxide Impregnated Carbon Nanotubes: Kinetics and Isotherms Study

**DOI:** 10.1155/2017/2853925

**Published:** 2017-03-12

**Authors:** Aamir Abbas, Basim Ahmed Abussaud, Ihsanullah, Nadhir A. H. Al-Baghli, Halim Hamid Redhwi

**Affiliations:** ^1^Department of Chemical Engineering, King Fahd University of Petroleum & Minerals, Dhahran 31261, Saudi Arabia; ^2^Department of Chemical Engineering, University of Engineering and Technology, Lahore 54890, Pakistan

## Abstract

Multiwall carbon nanotubes (CNTs) and iron oxide impregnated carbon nanotubes (CNTs-iron oxide) were investigated for the adsorption of hazardous toluene and paraxylene (p-xylene) from aqueous solution. Pure CNTs were impregnated with iron oxides nanoparticles using wet impregnation technique. Various characterization techniques including thermogravimetric analysis, scanning electron microscopy, elemental dispersion spectroscopy, X-ray diffraction, and nitrogen adsorption analysis were used to study the thermal degradation, surface morphology, purity, and surface area of the materials. Batch adsorption experiments show that iron oxide impregnated CNTs have higher degree of removal of p-xylene (i.e., 90%) compared with toluene (i.e., 70%), for soaking time 2 h, with pollutant initial concentration 100 ppm, at pH 6 and shaking speed of 200 rpm at 25°C. Pseudo-second-order model provides better fitting for the toluene and p-xylene adsorption. Langmuir and Freundlich isotherm models demonstrate good fitting for the adsorption data of toluene and p-xylene.

## 1. Introduction

Toluene and p-xylene are produced in different refinery operations and widely utilized in different petrochemical industries as a raw material. Toluene is used as a solvent in paints, cleaners, and degreasers and can also be utilized for surface coatings. It is also used as a raw material in explosives and polyurethanes production. Xylene exists as a clear liquid and can be found in three different isomeric forms: orthoxylene (o-xylene), metaxylene (m-xylene), and paraxylene (p-xylene). It has applications as a solvent in paints removers, cleaners, and inks. P-xylene is also used in the manufacturing of terephthalic acid (PTA), a feed stock for the production of polyester resins [1, 2].

Toluene and p-xylene are hazardous chemicals for human beings and environment. They have a number of harmful effects on human health including kidney, liver, and nervous system damage [3]. It is important to remove these hazardous compounds from the water before discharging from the facility. Removal of toluene and p-xylene was investigated heavily in the literature [4–9]. Among various methods, adsorption is the most economical, suitable, and widely practiced method for the removal of toluene, p-xylene, and other hydrocarbons from water. Researchers are in quest of the novel adsorbents with the improved adsorption capacity, high removal efficiency, easy regeneration, and handling capabilities [10, 11]. In recent years CNTs [12], a new class of materials, were introduced with high adsorption capacity and removal efficiency for removal of different organic, inorganic, and biological contaminants from water [5, 10, 11, 13–17].

CNTs have good surface modification ability and high surface area that is advantageous in many adsorption applications. CNTs modification with different functional groups resulted in higher removal efficiency of toluene and p-xylene [11, 18–22]. Metal oxide nanoparticles impregnated CNTs exhibited excellent adsorption capacity and efficiency for the removal of a number of contaminants from water [23–28].

In the present study, pure and CNTs impregnated with iron oxide nanoparticles were used for the adsorption of toluene and p-xylene from water. The synthesized materials were characterized using various material characterization tools. Batch adsorption experiments were performed and the effects of contact time, adsorption dosage, and initial concentration of adsorbate were determined on the removal of toluene and p-xylene from water. The kinetics of toluene and p-xylene were analyzed using pseudo-first-order, second-order, and intraparticle diffusion model. Adsorption isotherm studies of toluene and p-xylene were carried out using Langmuir, Freundlich, and Dubinin-Radushkevich (D-R) isotherm models.

## 2. Materials and Methods

### 2.1. Materials Synthesis

Multiwall carbon nanotubes (CNTs) with 95% purity were purchased from Chengdu Organic Chemicals Co. Ltd. (China). Iron (III) nitrate nonahydrate, Fe (NO_3_)_3_·9H_2_O (Reagent grade Sigma-Aldrich, purity ≥ 98%), toluene, and p-xylene of analytical grade were purchased from Sigma-Aldrich. All chemicals were used with same purity as received. Pure CNTs were impregnated with iron oxide nanoparticles using wet impregnation technique. 18 g (90% wt. of CNTs and 10% wt. iron nitrate) of CNTs was immersed in 500 mL of ethanol (ACS spectrophotometric grade, 95.0%, Sigma-Aldrich) and the mixture was sonicated using a probe type sonicator (VCX-750, Sonics & Materials, CT, USA) for deagglomeration and proper distribution inside ethanol solvent. 2 g of iron nitrate salt was also dissolved in 100 mL ethanol and the resultant solution was added to CNTs dropwise and sonicated for proper mixing with CNTs. Solution was heated at 80–90°C in an oven overnight to evaporate the ethanol. On complete drying, sample was calcined in a furnace at 350°C for 4 hours.

### 2.2. Materials Characterization

Pure and impregnated CNTs were characterized using various techniques. In order to perform morphology and elemental analysis, samples were coated with 5 nm thick layer of platinum using Quorum sputter coater (Model: Q150R S). Scanning electron microscope (SEM Model: TESCAN MIRA 3 FEG-SEM) was used to analyze the morphology and structure of pure and iron oxide impregnated CNTs. Energy dispersive X-ray (EDX) was used to perform the elemental analysis of materials. Samples were also analyzed using transmission electron microscope (TEM Model JEOL JEM-2100F) to get the information about dispersion of nanoparticles on the surface of CNTs. It also provided the information about catalyst particles used for growing CNTs. TA Instrument (Model: SDTQ600) was applied for thermogravimetric analysis (TGA) of pure and impregnated CNTs. Samples were heated to 900°C, at heating rate of 10°C/min and air flow rate of 100 mL/min. This analysis performed under air provided the purity and thermal degradation of materials. X-ray diffraction (XRD) measurements of the materials were performed using XRD (Model: Bruker D8 Advance) equipped with Cu K*α* radiation source (40 kV, 20 mA) and operated at a scanning rate of 1° min^−1^ over 2*θ* range of 10–80°. XRD provided the information about the presence of different phases in materials. Nitrogen adsorption desorption was carried out at 77 K for determining the surface area and porosity of the materials using an automatic volumetric adsorption analyzer (Model: ASAP 2020, Micromeritics, USA). In this analysis, samples were degassed at 300°C under vacuum, prior to adsorption desorption isotherm measurement. The surface area (*S*_BET_) of the synthesized materials was calculated, based on the Brunauer-Emmett-Teller (BET) isotherm. Total pore volume and pore size distribution of the materials were determined by applying the Barrett-Joyner-Halenda (BJH) model to the adsorption isotherms [29, 30].

### 2.3. Toluene and p-Xylene Adsorption Experimentation

All adsorption experiments were performed in 125 mL glass flasks containing 50 mg of adsorbent and 100 mL solution. Samples were shaken on mechanical shaker (Lab Companion Model: SK-600) at 200 rpm and 25 ± 2°C. All solutions were prepared in deionized water. Blank experiments without adding adsorbent were also carried out to confirm the adsorption on glass walls and loss due to volatilization. After shaking, the samples were filtered using filter paper of 0.45 *μ*m pore size and analyzed. To study the effect of adsorbent amount, various amounts of adsorbent ranging from 25 to 150 mg were added to each flask containing 100 mL solution of toluene or p-xylene with initial concentration of 100 ppm. To investigate the kinetics of toluene and p-xylene adsorption, each glass flask containing 50 mg of adsorbent was filled with 100 mL of a 100 ppm toluene or p-xylene solution at 25 ± 2°C and placed on shaker. At regular time intervals, the samples were filtered, and concentration was analyzed. For adsorption isotherms data, 100 mL samples of toluene solution of different initial concentration (20–150 ppm) were treated with 50 mg of adsorbents. Similarly, for p-xylene 100 mL samples of different initial concentration (20–100 ppm) were treated with 50 mg of adsorbents. Initial and final concentrations of toluene and p-xylene were analyzed using gas chromatograph (Model: 7890B, Agilent Corp., USA) with flame ionization detection (GC-FID) and chemical oxygen demand (COD: HACH Model DR 3900) analyzer.

For the analysis using GC-FID, 1 *μ*L sample was injected in wax column (30 m length, 20 mm internal diameter). Temperature was raised from 40 to 100°C with ramp of 10°C/minute. Temperature of both the injection point and FID detector was 250°C. For COD analysis, 2 mL solution of toluene or p-xylene was added to ready-made vial solution and heated at 150°C for 2 hours using the furnace (HACH Model DRB 200). On completion of digestion, vials were cooled at room temperature and COD was analyzed using the spectrophotometer (HACH Model DR 3900). COD was converted to concentration using (1) and (2) for toluene and p-xylene, respectively:

(1) Concentration of toluene mg/L=COD of sample3.13

(2) Concentration of p-xylene mg/L=COD of sample3.16.

Percentages of removal and adsorption capacity were calculated using (3) and (4), respectively:

(3) Removal efficiency %=Co−CtCo∗100

(4) Adsorption capacity q=Co−CtVm,

where “*C*_*o*_” is the initial concentration (ppm) at the start of the experiment (*t* = 0), while “*C*_*t*_” is the concentration at time “*t*”. “*V*” is the volume (L) of the solution and “*m*” represents the amount (g) of the adsorbent dosage.

## 3. Results and Discussion

### 3.1. Characterizations of CNTs

#### 3.1.1. Scanning Electron Microscopy

**Figure 1:**
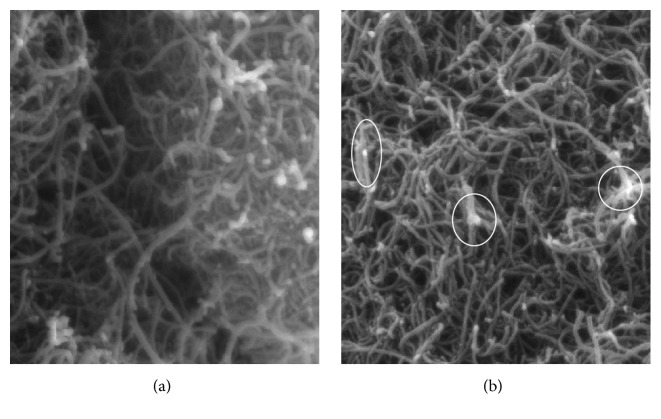
SEM images of (a) pure and (b) iron oxide impregnated CNTs conditions (voltage: 15 kV, resolution: 64 kX, and view field: 3 *μ*m).

Figure 1 shows the SEM images for pure and iron oxide impregnated CNTs. Tubular geometry of both pure and iron oxide impregnated CNTs was observed and no damage was indicated in CNTs structures after impregnation. Iron oxide nanoparticles were observed in white circles in Figure 1(b). It can be seen that the dispersion of CNTs has improved after impregnation with iron oxide nanoparticles. Iron oxide nanoparticles might help to reduce the strong Van der Waals forces between CNTs, hence leading to their dispersion.

#### 3.1.2. Energy Dispersive X-Ray Spectroscopy

**Figure 2:**
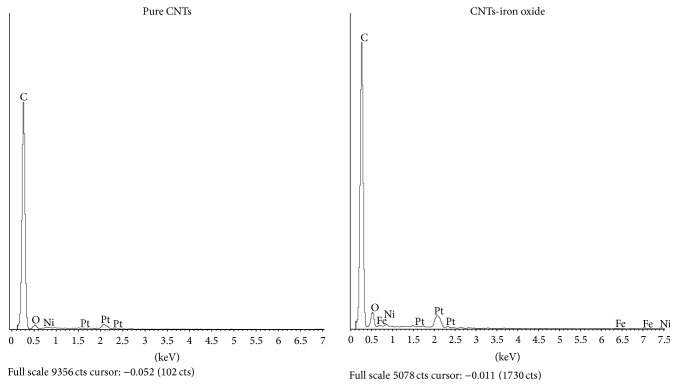
EDX analysis of pure and iron oxide impregnated CNTs.

Figure 2 demonstrates the EDX analysis of the materials. Analysis of the pure CNTs confirmed the presence of carbon as a main constituent. Presence of nickel was due to the catalyst particles used for growing CNTs, while platinum was used as a sputtering material. Analysis of iron oxide impregnated CNTs indicated the presence of iron in addition to the constituents of pure sample.

#### 3.1.3. Transmission Electron Microscopic Analysis

**Figure 3:**
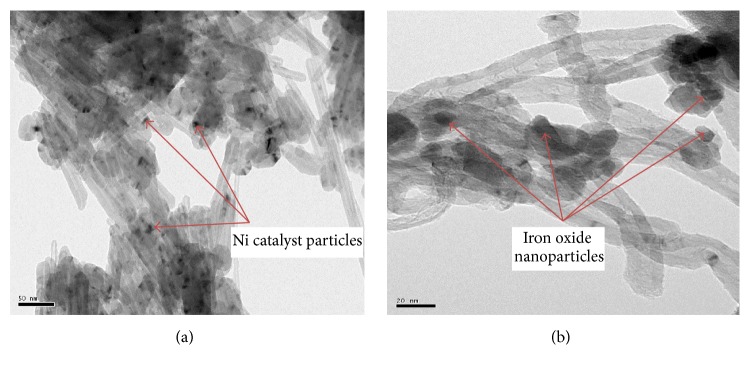
TEM images of (a) pure and (b) iron oxide impregnated CNTs.

Figure 3 provides the TEM images for both pure and iron oxide impregnated CNTs. Highly well-ordered crystalline structure of multiwall carbon nanotubes was observed in Figure 3(a). Nickel particles used for growing CNTs were also observed in the image and indicated with arrows.  Figure 3(b) provides the distribution of iron oxide nanoparticles on the surface of CNTs. Small and irregular shaped iron nanoparticles were observed in the sample. It was also observed that particles are widely distributed on the surface of CNTs with diameter range of 5–10 nm. At some locations particles also seem agglomerated making clusters.

#### 3.1.4. Thermogravimetric Analysis

**Figure 4:**
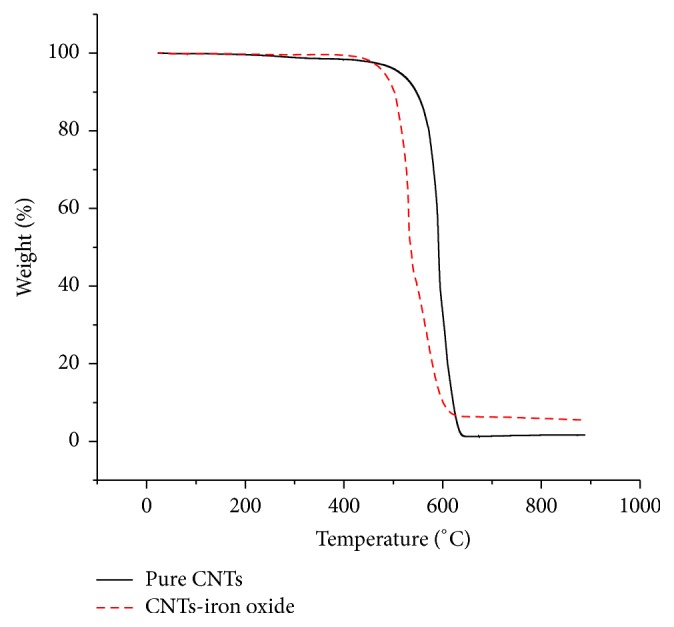
TGA plot of pure and iron oxide impregnated CNTs.

Figure 4 indicates the TGA for both pure and iron oxide impregnated CNTs. Both of the TGA curves have two main weight loss regions. Initial small weight loss of around 2% was attributed to the evaporation of physically bound water and some other lighter impurities. The second, steep and rapid weight loss region represents the combustion of CNTs. Pure CNTs showed more stability and started degrading around 550°C while degradation of iron oxide impregnated CNTs started around 500°C. This may be due to the fact that the impregnation of iron oxide nanoparticles on CNTs serves as an impurity, hence leading to steep weight loss at lower temperature [31]. Additionally, iron oxide nanoparticles reduced the agglomeration of CNTs as shown in SEM images that might also led to easy degradation [32]. Around 1% weight of the material was left at the end of the analysis for pure CNTs. This indicated the presence of nickel nanoparticles that were used as a catalyst for synthesis of CNTs. Iron oxide impregnated CNTs showed higher weight residue of around 7%, which represent the weight of iron oxide nanoparticles in addition to the nickel catalyst.

#### 3.1.5. X-Ray Diffraction

**Figure 5:**
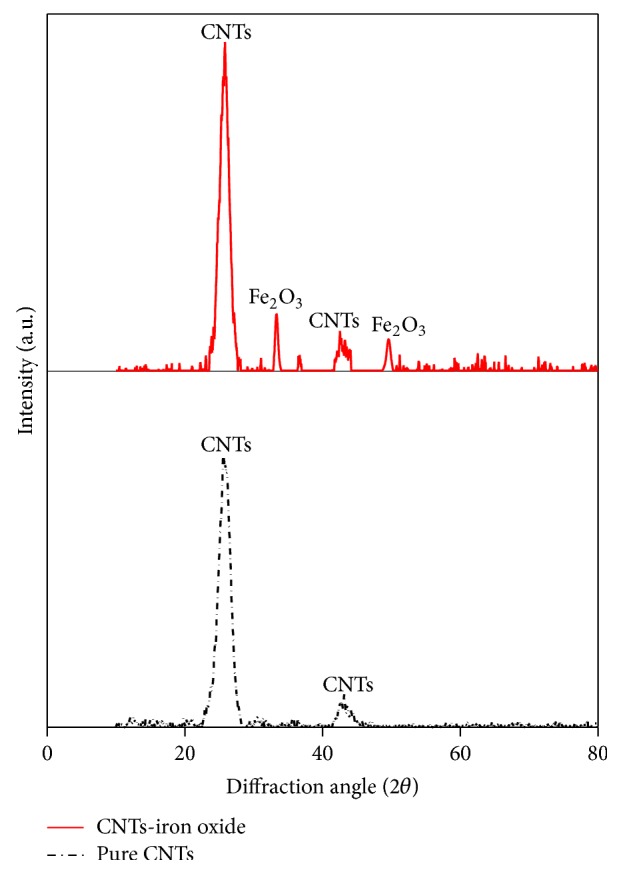
XRD analysis of pure CNTs and iron oxide impregnated CNTs.

Figure 5 shows the XRD pattern of the pure and iron oxide impregnated CNTs. XRD pattern of iron oxide impregnated CNTs showed additional peaks, when compared with XRD pattern of pure CNTs. The characteristic peaks of graphite carbon were seen in both samples at 2*θ* of 26° and 43° that represented the presence of CNTs. Additional peaks of iron oxide in impregnated CNTs sample are indicated by the representative peaks at 2*θ* of 35° and 52° [33].

#### 3.1.6. Surface Area and Pore Size Analysis

**Figure 6:**
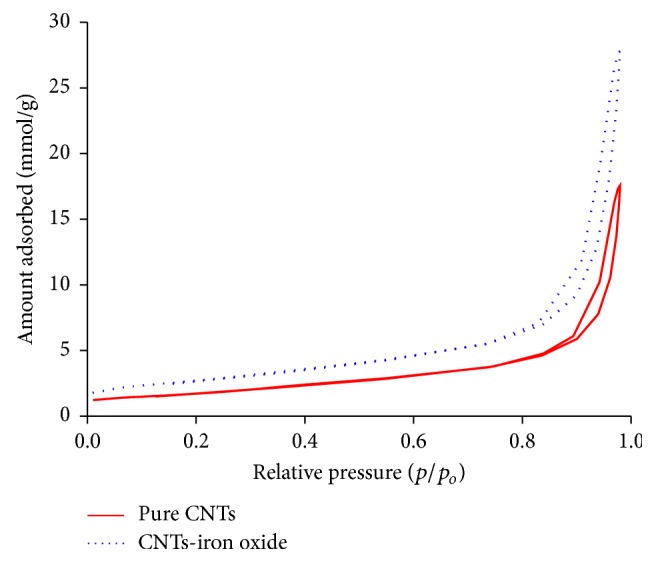
Nitrogen adsorption desorption isotherms for pure and iron oxide impregnated CNTs.

Nitrogen adsorption desorption isotherm curves for pure and iron oxide impregnated CNTs are shown in Figure 6 and classified as Type V according to international union of pure and applied chemistry (IUPAC) classification. Type V indicates the presence of mesopores and external sites for adsorption of molecules on the surface of pure and iron oxide impregnated CNTs. The hysteresis loop was found of type H3 in each curve and occurred due to capillary condensation [34].  Table 1 provides the BET surface area of the pure and iron oxide impregnated CNTs. It was observed that the iron oxide impregnated CNTs have higher surface area (216 m^2^/g) compared with pure CNTs (138 m^2^/g). This increase in surface area of the iron oxide impregnated CNTs might be due to improved distribution and deagglomeration of CNTs after attachment of iron oxide nanoparticles which is in accordance with Type V assumptions of mesopores and external surface availability for adsorption. Mean pore size indicates the mesopores for pure and iron oxide impregnated CNTs. Based on the results presented in Table 1, total pore volume was 0.61 cm^3^/g for pure CNTS and 0.96 cm^3^/g for iron oxide impregnated CNTs. This higher surface area and pore volume of iron oxide impregnated CNTs may be useful for adsorption. Mean pore radius was found in the range of mesopores for both materials.

### 3.2. Adsorption Experimentation Results

#### 3.2.1. Effect of Contact Time

**Figure 7:**
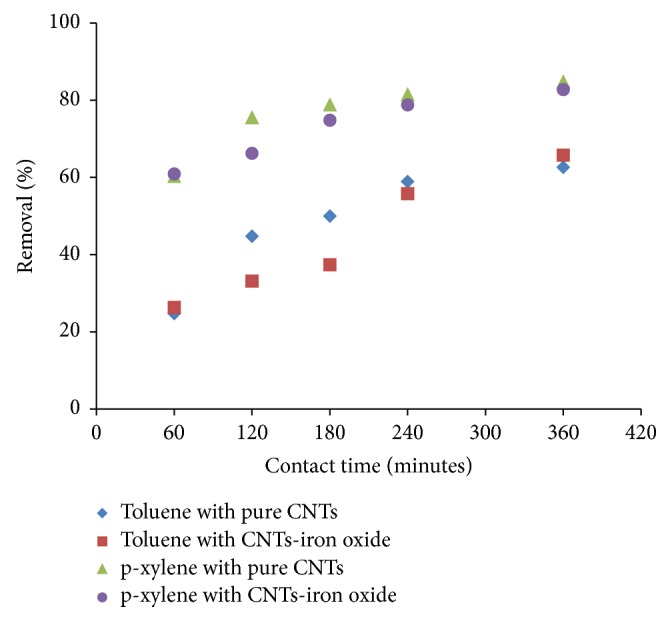
Effect of contact time on the removal of toluene and p-xylene (initial concentration: 61 mg/L for toluene and 48 mg/L for p-xylene, adsorbent dosage: 50 mg, shaking speed: 200 rpm, pH: 6, and temperature: 298 K).

Figure 7 provides the effect of contact time on the removal of toluene and p-xylene using the pure and iron oxide impregnated CNTs. The removal efficiency enhanced with increasing contact time for both pure and and iron oxide impregnated CNTs until equilibrium was attained. Initially, higher removal was due to plenty of active sites available that contributed to fast removal of adsorbate molecules. With the passage of time, the number of vacant active sites reduced and removal was observed to decrease. Furthermore, layers of the adsorbed molecules offer additional resistance to the new molecules to penetrate through.

Removal of p-xylene was found to be higher compared with toluene under same experimental conditions, except initial concentration of 61 ppm for toluene and 48 ppm for p-xylene. This can be attributed to low solubility and higher hydrophobicity of p-xylene compared with toluene. Solubility of toluene is 530 mg/L in water while p-xylene has solubility of 150.5 mg/L. Generally, the decrease in solubility for hydrophobic organic compounds (hydrophobicity based on log⁡*K*_ow_ is 2.69 for toluene and 3.15 for p-xylene) leads to increase in adsorption. Similar trends were reported for adsorption of benzene, toluene, ethylbenzene, and p-xylene using various adsorbents in some studies [18, 36]. Furthermore, it was observed that percentage removal efficiency was almost similar using pure and iron oxide impregnated CNTs for both toluene and p-xylene after 240 minutes.

#### 3.2.2. Effect of Adsorbent Amount

**Figure 8:**
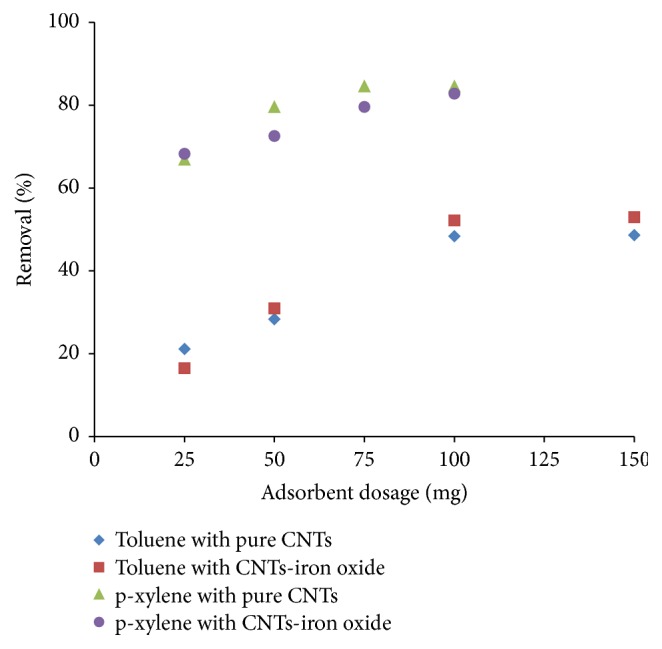
Effect of the adsorbent amount on removal of toluene and p-xylene (initial concentration: 61 mg/L for toluene and 48 mg/L for p-xylene, contact time: 2 hr, shaking speed: 200 rpm, pH: 6, and temperature: 298 K).

Figure 8 provides effect of the adsorbent amount on the removal of both contaminants. It is obvious that with increasing amount of the adsorbent, removal of both the toluene and p-xylene increased. Higher quantity of the adsorbent provided more adsorption sites, hence leading to higher removal of the contaminants. As adsorbent dosage was increased from 25 to 100 mg, removal increased from 21 to 48% and from 16 to 52% for toluene using pure and iron oxide impregnated CNTs, respectively. Similarly, for p-xylene, by increasing the adsorbent amount from 25 mg to 75 mg, removal increased from 66 to 84% and from 68 to 80% for pure and iron oxide impregnated CNTs, respectively. Further increase in adsorbent amount does not affect much removal efficiency because it achieved the equilibrium adsorption capacity. Similar findings were also reported elsewhere [37]. Although surface area and pore volume were higher for iron oxide impregnated CNTs but it was found that the removal efficiency of both pure and impregnated CNTs was almost similar for the adsorption of toluene and p-xylene. With the same amount of the adsorbent, p-xylene showed higher removal percentage compared with toluene which was due to lower solubility and higher hydrophobicity of p-xylene.

#### 3.2.3. Adsorption Kinetics Study

Adsorption kinetic is one of the most important factors that govern the solute uptake rate and represents the adsorption efficiency of the adsorbent. Pseudo-first-order, second-order, and Weber-Morris intraparticle diffusion model were used for the kinetics model fitting of toluene and p-xylene adsorption data. Representative equations of these models are provided below.

*The Pseudo-First-Order Model*

(5) $$\begin{array}{c} \ln\left(\;q_{e}-q_{t}\;\right)=\ln\left(\;q_{e}\;\right)-k_{1}t. \end{array}$$

*The Pseudo-Second-Order Model*

(6) $$\begin{array}{c} \frac{t}{q_{t}}=\frac{1}{k_{2}q_{e}^{2}}+\frac{t}{q_{e}}. \end{array}$$

**Figure 9:**
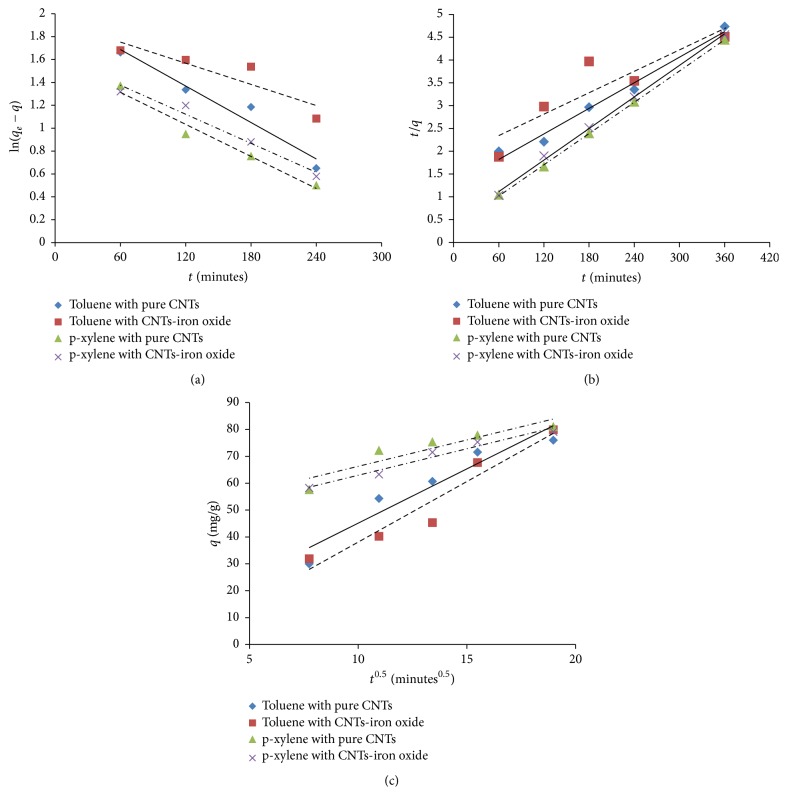
Adsorption kinetics model fitting using (a) pseudo-first-order, (b) pseudo-second-order, and (c) intraparticle diffusion model for toluene and p-xylene.

*The Weber-Morris Intraparticle Diffusion Model*

(7) $$\begin{array}{c} q_{t}=k_{id}t^{0.5}+C, \end{array}$$

where *q*_*t*_ and *q*_*e*_ are the concentrations of contaminants on adsorbent at time “*t*” and equilibrium, respectively. *k*_1_ is pseudo-first-order model constant, *k*_2_ is second-order model constant, and *k*_id_ is intraparticle diffusion model.  Figure 9 indicates the fitting of experimental data with kinetics models for toluene and p-xylene.

Table 2 provides the results of the kinetics model fittings for the adsorption of toluene and p-xylene using pure and iron impregnated CNTs. It was observed that pseudo-second-order model was best to describe the adsorption of toluene and p-xylene using pure and iron impregnated CNTs. The values of regression coefficient (*R*^2^) were highest for pseudo-second-order model ranging from 95 to 97% except for toluene adsorption using iron oxide impregnated CNTs with value of 80%. The experimentally calculated values of adsorption capacities were in good combination with the values obtained from pseudo-second-order model fitting. It was also noted that fitting of data using intraparticle diffusion model was linear but does not pass through the origin; therefore intraparticle diffusion is not a sole rate controlling step. Therefore, the overall adsorption kinetics might be dependent on the boundary layer diffusion in addition to the intraparticle diffusion. Similar trends were reported elsewhere [38].

It can be observed that p-xylene has higher values of the constants. This might be due to the introduction/presence of additional methyl groups in the p-xylene which may help in faster removal. One more interesting observation was that percentage removal of p-xylene was higher compared with toluene but the adsorption capacities are lower for p-xylene using both adsorbents, which was due to lower initial concentration of p-xylene used for kinetics data as described in Section 3.2.1.

#### 3.2.4. Adsorption Isotherms Study

Adsorption equilibrium data of the toluene and p-xylene using pure and iron oxide impregnated CNTs was fitted with Langmuir, Freundlich, and D-R isotherm model. These models have been widely used to study the adsorption of various adsorbates on CNTs. Nonlinear forms of these models were used to avoid the error due to linearization. Langmuir model best describes the monolayer adsorption while Freundlich model provides information about heterogeneous adsorption on adsorbent surface [39]. Representative equations of the isotherm models are presented below.

*Langmuir Isotherm Model*

(8) $$\begin{array}{c} q_{e}=\frac{q_{m}K_{L}C_{e}}{1+K_{L}C_{e}}. \end{array}$$

*Freundlich Isotherm Model*

(9) $$\begin{array}{c} q_{e}=K_{F}{C_{e}}^{1/n}. \end{array}$$

**Figure 10:**
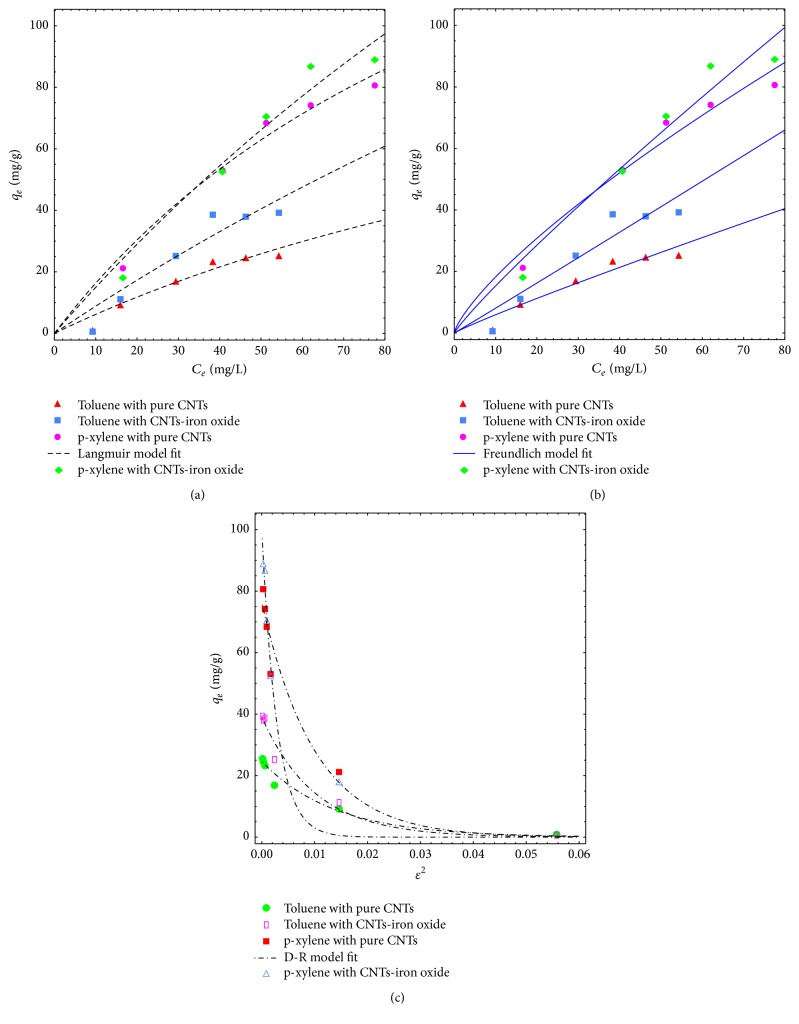
Adsorption isotherm model fitting using (a) Langmuir, (b) Freundlich, and (c) D-R model for toluene and p-xylene.

*D-R Isotherm Model*

(10) $$\begin{array}{c} q_{e}=q_{m}e^{-B\varepsilon^{2}}, \end{array}$$

where *C*_*e*_ and *q*_*e*_ are the concentrations of contaminants in water and in adsorbent at the adsorption equilibrium, respectively. *q*_*m*_ is the maximum adsorption capacity; *K*_*L*_ is the adsorption equilibrium constant of Langmuir model; *K*_*F*_ and *n* are Freundlich constants related to the adsorption capacity and surface heterogeneity of the adsorbents, respectively.  Figure 10 represents the fitting of data with isotherm models while adsorption parameters and regression data of the models are presented in Table 3. Regression coefficient (*R*^2^) has almost equal value for the Langmuir and Freundlich model for the adsorption of both toluene and p-xylene on pure and impregnated CNTs. It is evident from the results that values of rate constants *K*_*L*_ and *K*_*F*_ were higher for p-xylene compared with values for toluene, which can be attributed to low solubility and higher hydrophobicity of p-xylene. Lower solubility of p-xylene in water might be helpful in providing more attraction towards CNTs surface and fast adsorption rate. Values of “*n*” are close to 1 in all cases which indicates the suitable and uniform adsorption of toluene and p-xylene. Activation energy “*E*_*a*_” was calculated using D-R isotherm model fitting. It was found that values are less than 1 which indicates physical adsorption of toluene and p-xylene molecules on the surface of adsorbents. This phenomenon can be helpful in easy regeneration of adsorbents for reuse.

#### 3.2.5. Comparison with Existing Literature

Comparison of adsorption capacity for removal of toluene and p-xylene using single wall carbon nanotubes (SWCNTs), multiwall carbon nanotubes (CNTs), and modified CNTs is shown in Table 4. It is observed from the results of Table 4 that iron oxide impregnated CNTs have relatively higher adsorption capacity compared with other adsorbents previously reported in literature. CNTs impregnated with iron oxide can be good adsorbent for removal of toluene and p-xylene from large volume of water.

## 4. Conclusions

Wet impregnation technique was used for synthesizing iron oxide impregnated CNTs. Materials were characterized using SEM, EDX, TGA, XRD, and nitrogen adsorption desorption analysis. Removal of toluene and p-xylene was carried out in batch experiments and effect of contact time, adsorbent amount, and initial concentration was studied. Results demonstrate higher removal of p-xylene compared with toluene under almost similar experimental conditions. Kinetic studies show that adsorption of toluene and p-xylene obeys a pseudo-second-order model. Adsorption isotherms study indicated that Langmuir and Freundlich isotherm models demonstrate very good fit with experimental data. Adsorption capacity of p-xylene was calculated using Langmuir model fit as 219 mg/g and 458 mg/g for pure and iron oxide impregnated CNTs while it was 127 mg/g and 381 mg/g for toluene adsorption using pure and iron oxide impregnated CNTs.

## Figures and Tables

**Table 1:** Surface and structural parameters of pure and iron oxide impregnated CNTs.

Physical property	Materials	
	Pure CNTs	CNTs-iron oxide
BET surface area (m^2^/g)	138	216
Total pore volume (cm^3^/g)	0.61	0.96
Cumulative pore area (m^2^/g)	145	207
Mean pore radius (A°)	167	185

**Table 2:** Adsorption kinetics parameters of toluene and p-xylene adsorption.

Model	Parameters	Toluene		p-xylene	
		Pure CNTs	CNTs-iron oxide	Pure CNTs	CNTs-iron oxide
	*C*_*o*_	60.70	60.70	47.78	47.78

Experimental	*q*_*e*,experimental_	76.04	79.87	81	79.11

Pseudo-first-order	*k*_1_(min^−1^)*∗*10^−3^	5.3	3.1	4.7	4.2
	*q*_*e*,calculated_	7.43	6.94	4.92	5.10
	*R* ^2^ (%)	95	80	97	97

Pseudo-second-order	*k*_2_ (g mg^−1^ min^−1^)*∗*10^−4^	0.69	0.32	3.9	3.1
	*q*_*e*,calculated_	107.53	128.21	87.72	86.96
	*R* ^2^ (%)	98	80	99.9	99.7

Intraparticle diffusion model	*k*_id_ (g mg^−1^ min^−0.5^)	4.04	4.51	1.96	1.97
	*C*	4.74	−6.99	46.66	43.23
	*R* ^2^ (%)	91	93	85	96

**Table 3:** Isotherm models parameters for toluene and p-xylene adsorption.

Model	Parameters	Toluene		p-xylene	
		Pure CNTs	CNTs-iron oxide	Pure CNTs	CNTs-iron oxide
Langmuir	*K*_*L*_ (L/mg)	0.005	0.002	0.008	0.003
	*q*_*m*_ (mg/g)	127.94	381.18	219.51	458.52
	*R* ^2^ (%)	98.5	97.6	99.7	99.4

Freundlich	*K*_*F*_ (mg/g)/(mg/L)^*n*^	0.71	0.79	3.18	1.93
	*n*	1.08	0.99	1.32	1.11
	*R* ^2^ (%)	98.3	97.6	99.5	99.3

Dubinin-Radushkevich (D-R)	*q*_*m*_ (mg/g)	24.62	39.11	76.32	100.58
	B (mole^2^/kJ^2^)	72.87	99.73	99.81	349.98
	*E*_*a*_ (kJ/mole)	0.08	0.07	0.07	0.04
	*R* ^2^ (%)	99.2	99.2	99	98.4

**Table 4:** Comparison of adsorption capacity of different CNTs based adsorbents for toluene and p-xylene removal.

Adsorbent	Adsorption capacity (mg/g)		Conditions	References
	Toluene	p-xylene		
CNT (NaOCl)	279.8	413.77	pH 7, T 298 K	[20]
SWCNT	—	77.5	pH 5.4, T 298 K	[22]
SWCNT (HNO_3_)	—	85.5	pH 5.4, T 298 K	[22]
CNTs-KOH	87.12	—	pH 7, T 293 K	[19]
CNT	80.1	147.8	pH 7, T 298 K	[35]
CNT (NaOCl)	252.1	318.3	pH 7, T 298 K	[35]
Pure CNTs	127.94	219.51	pH 7, T 298 K	This work
CNTs-iron oxide	381.18	458.52	pH 7, T 298 K	This work
